# Sleep and Dietary Patterns in Pregnancy: Findings from the GUSTO Cohort

**DOI:** 10.3390/ijerph14111409

**Published:** 2017-11-17

**Authors:** Linde van Lee, Ai-Ru Chia, See Ling Loy, Marjorelee Colega, Elaine K. H. Tham, Shirong Cai, Fabian Yap, Keith M. Godfrey, Oon Hoe Teoh, Daniel Goh, Kok Hian Tan, Yap-Seng Chong, Birit F. P. Broekman, Mary F. F. Chong

**Affiliations:** 1 Singapore Institute for Clinical Sciences, Agency for Science, Technology and Research, Singapore 117549, Singapore; Linde_van_Lee@sics.a-star.edu.sg (L.v.L.); marjorelee_colega@sics.a-star.edu.sg (M.C.); Elaine_Tham@sics.a-star.edu.sg (E.K.H.T.); yap_seng_chong@nuhs.edu.sg (Y.-S.C.); Birit_Broekman@sics.a-star.edu.sg (B.F.P.B.); 2 Department of Obstetrics and Gynaecology, Yong Loo Lin School of Medicine, National University of Singapore, Singapore 119074, Singapore; chiaairu@u.nus.edu (A.-R.C.); obgcais@nus.edu.sg (S.C.); 3 Duke-NUS Medical School, Singapore 169857, Singapore; loy.see.ling@kkh.com.sg (S.L.L.); fabian.yap.kp@kkh.com.sg (F.Y.); 4 Department of Reproductive Medicine, KK Women’s and Children’s Hospital, Singapore 229899, Singapore; tan.kok.hian@kkh.com.sg; 5 Department of Pediatrics, KK Women’s and Children’s Hospital, Singapore 229899, Singapore; teoh.oon.hoe@singhealth.com.sg; 6 Lee Kong Chian School of Medicine, Nanyang Technological University, Singapore 308232, Singapore; 7 MRC Lifecourse Epidemiology Unit, University of Southampton, Southampton SO16 6YD, UK; kmg@mrc.soton.ac.uk; 8 NIHR Southampton Biomedical Research Centre, University Hospital Southampton NHS Foundation Trust, Southampton SO16 6YD, UK; 9 Department of Pediatrics, Yong Loo Lin School of Medicine, National University of Singapore, Singapore 119228, Singapore; daniel_goh@nuhs.edu.sg; 10 Department of Psychiatry, VU Medical Centre, 1081 HV Amsterdam, The Netherlands; 11 Saw Swee Hock School of Public Health, National University of Singapore, Tahir Foundation Building, 12 Science Drive 2, #09-01Q, Singapore 117549, Singapore; 12 Clinical Nutrition Research Center, Agency for Science, Technology and Research, Singapore 117609, Singapore

**Keywords:** dietary patterns, diet quality, sleep quality, sleep duration, eating behaviors, pregnancy

## Abstract

Evidence on the association between sleep, diet, and eating behaviors in pregnant women is lacking. We examine this in a cohort of apparently healthy pregnant women. At 26–28 weeks gestation, 497 participants completed the Pittsburgh Sleep Quality Index to assess sleep and a 24-h recall to assess dietary intake. Diet quality was assessed by the Healthy Eating Index for pregnant women in Singapore (HEI-SGP) score and previously derived dietary patterns (vegetables-fruit-rice, seafood-noodles, and pasta-cheese-meat pattern). Eating behaviors studied included the longest night-time fasting interval, frequency of consumption occasions, energy from discretionary foods, and nighttime eating. Adjusted means were estimated between poor/good quality and short/normal sleepers using linear regressions, including covariates. Good sleep quality versus poor sleep quality, was associated with better diet quality (mean HEI-SGP 54.6 vs. 52.0; *p* = 0.032), greater adherence to the vegetables-fruit-rice pattern (mean 0.03 vs. −0.15; *p* = 0.039), lesser adherence to the seafood-noodle pattern (mean −0.14 vs. 0.03; *p* = 0.024), and a trending lower calories from discretionary foods (mean 330.5 vs. 382.6 kcal; *p* = 0.073), after adjusting for covariates. After additional adjustment for anxiety, only sleep quality and the seafood-noodle pattern remained significantly associated (*p* = 0.018). Short sleep was not associated with any diet or eating behavior. In conclusion, good sleep quality is associated with a better diet quality and a greater adherence to the vegetable-fruit-rice pattern, but with lesser adherence to the seafood-noodle diets in pregnant women.

## 1. Introduction

Dietary intake and thereby the risk of chronic diseases and excessive weight gain can be altered by many lifestyle behaviors. Evidence is accumulating that sleep is one of these factors [1,2,3]. Shorter sleep, in particular, may affect dietary intake through a number of possible mechanisms [3,4]; shorter sleep gives more time and opportunities for eating, it may change the timing of eating, and may induce hedonic feeding. Also, it has been suggested that short sleep alters the release of the appetite-related hormones. Studies have observed lower leptin and higher ghrelin concentrations, which are associated with increased dietary intake, in short sleepers [4]. 

Most research on sleep and dietary intake has focused on macronutrients or single nutrient intakes [4], whereas examining the diet holistically has been suggested to better reflect the complexity of dietary intake [5]. The two most common methods to study overall diet are diet quality indices that are based on pre-existing knowledge of diet-disease associations (e.g., dietary recommendations) or data-reductions methods (e.g., principal components analyses) [6]. To date, evidence from trials in adults and children suggests that sleep restriction increases food and total energy intakes [1]. Moreover, short sleep duration (<6–7 h) as compared to adequate sleep (7–8 h) was associated with poorer diet quality as assessed with diet quality scores in Canadian children [7], European adolescents [8], and Iranian adult women [9]. According to another review, mainly including cross-sectional studies, diets that were high in fat were associated with more sleep disorders, whereas Mediterranean-style diets and diets high in carbohydrates were associated with fewer insomnia symptoms [10].

Beyond dietary intake itself, sleep might also alter eating behaviors possibly leading to concurrent changes in dietary intake and the risk of chronic disease. It has been reported that sleep deprivation or short sleep duration (<6 h) increases portion size, the likelihood of main meal skipping, and was associated with greater energy intake from snacks and longer eating periods [11,12,13,14]. 

Sleep quality and sleep duration are often compromised in pregnant women and deteriorate over the course of pregnancy [15,16], mainly due to physical discomfort and pain [17]. Sleep behavior may, therefore, be an important risk factor of unhealthy dietary practices during pregnancy and creates a window for development of public health education programs. We are aware of only one study that investigated sleep quality and dietary intake among pregnant women. Chang et al. showed positive significant associations between night time sleep disturbance and dietary fat intake and between shorter time to fall asleep, and higher fruit and vegetables intake in overweight and obese American women during pregnancy [18]. However, diet quality or dietary patterns were not studied. Thus, it is yet unknown whether sleep is associated with dietary patterns and eating behaviors during pregnancy. Hence, the aim of this study is to understand the association between dietary patterns and eating behaviors in apparently healthy pregnant women using the Growing Up in Singapore Towards Healthy Outcomes (GUSTO) cohort. 

## 2. Methods

### 2.1. Study Design and Population

The GUSTO mother-offspring cohort included first-trimester pregnant women aged between 18 and 50 years old residing in Singapore between June 2009 and September 2010 [19]. Inclusion criteria were having the intention to deliver in the two major study hospitals in Singapore, agree to donate placenta, cord, and cord blood at delivery, and spouse had homogenous parental background of Chinese, Malaysian, or Indian descent. Women suffering from serious health conditions, such as type 1 diabetes mellitus, cancer, or psychological disorders were not eligible. Medical ethical approval was obtained from the National Healthcare Group Domain Specific Review Board (reference number D/09/021) and the Sing Health Centralized Institutional Review Board (reference number 2009/280/D) and all participants gave written informed consent.

### 2.2. Dietary Intake, Diet Quality and Dietary Pattern Analysis

One 24-h recall was used to assess dietary intake at 26–28 weeks of gestation at a clinic visit. This clinic visit was scheduled at this time point to, after the assessment of diet, also administer the oral glucose tolerance test, which is commonly administered at 24–28 weeks gestation. The 24-h recall was conducted face-to-face by trained research assistants using the five-stage multiple pass method. This method is a validated technique to increase the accuracy of recalls [20]. In addition to the dietary intake, the timing of all foods and drinks was recorded during the 24-h recall. Nutrient and energy intakes were calculated using the local food composition table [21], the USA national nutrient database [22], or information from food labels. 

The Healthy Eating Index for Pregnant women in Singapore (HEI-SGP) was used as the measure for diet quality [23]. This continuous index reflects adherence to the Singapore dietary guidelines for pregnant women, and comprises 11 components on total fruit, whole fruit, total vegetables, dark green leafy and orange vegetables, total rice and alternatives, whole grains, dairy, total protein foods, use of antenatal supplements, total fat, and saturated fatty acids. The fruit and vegetable component scores ranged from zero to five, whereas the remaining components had a maximum of ten points. The total summed score (0–90 points) was converted to a 0–100 scale for easier interpretation and higher scores correspond to greater adherence to the dietary recommendations. The HEI-SGP has been shown to be a useful tool to differentiate diet quality within the GUSTO population [23]. 

Dietary patterns were derived from all of the GUSTO participants with available dietary intake (*n* = 1170) [24]. All of the recorded food and drinks from the 24-h recall were categorized into 68 food groups according to their similarities in nutritional content and culinary usage. Exploratory factor analysis with Varimax rotation was used to obtain independent patterns. Dietary pattern scores rank individuals according to their degree to which they adhere to each dietary pattern, where higher scores mean greater adherence to that particular pattern. Each individual gets a score for each of the derived patterns. Based on the scree plot and the interpretability of the derived patterns, three dietary patterns were identified: the vegetable, fruit and white rice pattern (VFR), the seafood, fish, and noodle pattern (SFN), and the pasta, cheese, and processed meat pattern (PCP). The VFR pattern was characterized by high loadings for vegetables, fruits, and rice, and negative loadings for fried potatoes, hamburgers, and carbonated drinks. The SFN was characterized by high loadings for soup, seafood, fish and seafood products, noodles, and low-fat red meat, and low loadings for legumes, ethnic bread, white rice and curry-based gravies. The PCP was characterized by high loadings for pasta, cheese, processed meat and tomato, and cream-based gravies [24]. Factor loadings for the three dietary patterns can be found in Table S1, and further details on principal component analyses can be found elsewhere [24]. 

### 2.3. Eating Behaviors

Using the timing of intake reported during the 24-h recall, the longest night-time fasting interval was assessed from 19.00 h to 06.59 h. Frequency of eating was defined as the number of eating occasions that provided ≥50 kcal, with a time interval between eating occasions of ≥15 min [25]. Night-time eating was defined calculated as the percentage of energy intake during night time (19.00 h to 06.59 h) from total energy intake [26]. These three behaviors have previously been associated with 2-h and fasting glucose concentrations during pregnancy within the GUSTO cohort [25,26], suggesting that they can be considered as unfavorable eating behaviors. Lastly, energy from discretionary calories was defined as the sum of energy from caloric beverages (≥5 kcal; excluding plain water, diet soda, and unsweetened coffee, tea, and cow’s milk), local cakes, desserts, and snacks.

### 2.4. Sleep Quality and Sleep Duration Assessment

Sleep quality and sleep duration were measured using the self-administered Pittsburgh Sleep Quality Index (PSQI) [27] during the clinic visit at 26–28 weeks gestation. The PSQI contains 19 items that generate seven subcomponents scores (i.e., subjective sleep quality, sleep latency, sleep duration, habitual sleep efficiency, sleep disturbances, sleep medication, and daytime functioning) on a zero to three scale and a summed global score, ranging from 0 to 21. Higher scores represent poorer subjective overall sleep quality. Poor sleep quality was defined as a global PSQI score greater than five, which showed good sensitivity and specificity with insomnia in previous studies [27,28]. Those with poor sleep quality will be referred to as poor sleepers, whilst those with a global PSQI score of lesser than five will be referred to as good sleepers Sleep duration was estimated based on the question from the PSQI: “During the past month, how many hours of actual sleep did you get at night? (This may be different from the number of hours spend in bed)”. Short sleep was defined as <6 h following the guidelines of the national sleep foundation [29]. Excessive sleep (>10 h) was only observed in five participants, hence these were included in the normal-duration sleepers.

### 2.5. Covariates 

Participants’ characteristics (e.g., demographics, education level, household income, self-reported pre-pregnancy weight) and lifestyle behaviors (e.g., alcohol intake, smoking, physical activity level) were obtained by general questionnaires during recruitment and during the clinic visit at 26–28 weeks gestation [19]. Maternal height was measured with a SECA 213 stadiometer (SECA Corp., Hamburg, Germany) and was used to calculate body mass index (BMI, in kg/m^2^). Monthly household income was categorized as ≤S$1999, 2000–5999, ≥S$6000, and pre-pregnancy smoking (yes/no) was defined as smoking once a day for a year or longer. Physical activity during pregnancy was categorized as being moderate-to-strenuous active or not. Mental well-being was assessed by the Edinburgh Postnatal Depression Scale (EPDS) [19] and the State-Trait Anxiety Inventory (STAI) [22]. The EPDS rates the intensity of depressive symptoms in the past week and consists of 10-items scored between 0–3. EPDS scores ≥15 indicated probable major depression during pregnancy [20]. The STAI consists of two 20-item subscales and only the STAI-state was used in the present study because it reflects a transitory anxiety state instead of anxious personality traits. 

### 2.6. Statistical Analyses

**Figure 1:**
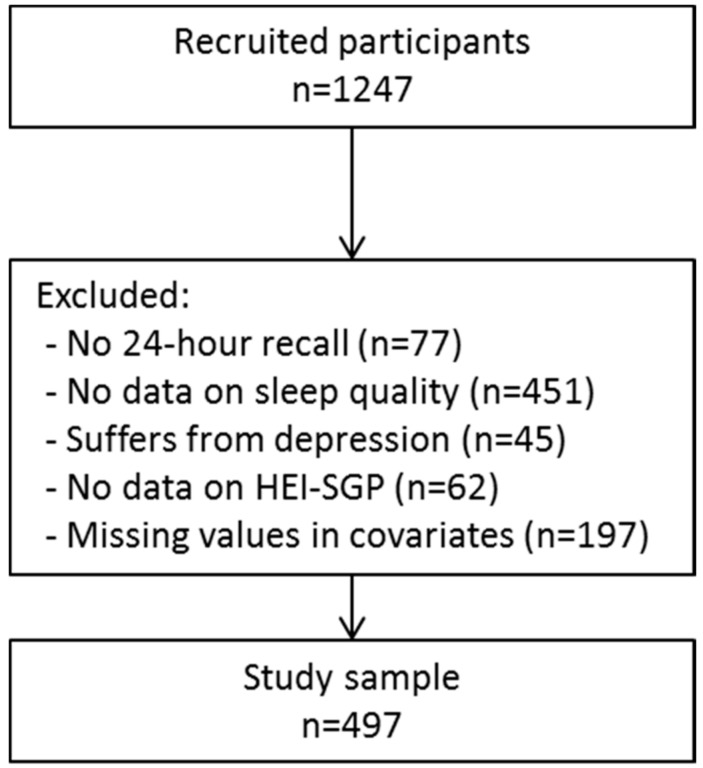
Flow chart of study participants. HEI-SGP: Healthy eating index for pregnant women in Singapore.

Of the 1247 recruited pregnant women, we excluded those with no information on dietary intake (*n* = 77), or sleep quality (*n* = 451). Furthermore, we excluded those with probable major depression (*n* = 45), because sleep problems can be a feature of depression [30] and those with missing values in the covariates (*n* = 197), resulting in a total study sample of 497 participants (Figure 1). As we excluded a considerable amount of participants without information on sleep quality and duration, we reran the principal components analyses with Varimax rotation to check whether similar dietary patterns emerged in this subsample. The newly emerged dietary patterns were similar, as indicated by similar food/food groups loading highly on the dietary patterns in similar extent, and by the moderate to strong correlation coefficients with previously obtained dietary patterns (r_VFR_ = 0.95, r_SFN_ = 0.94, r_PCP_ = 0.32; Table S1).

Descriptive analyses of the study sample were presented among poor and good sleepers. *p* for difference represents *t*-test for normally distributed continuous variables and Chi-square test for categorical variables. Multivariate linear regressions were used to estimate adjusted means of diets and eating behaviors between poor and good sleepers and short and normal sleepers while adjusting for alcohol intake during pregnancy (y/n), physical activity during pregnancy (y/n), household income (S$0–1999, S$2000–5999, ≥S$6000), education level (primary/secondary, post-secondary, university), ethnicity (Chinese, Malay, Indian), energy intake (kcal), age (y), and gravidity (1 child, >1 child). In the second model, we additionally adjusted for state anxiety as sleep and anxiety states are interrelated [30]. Case-wise exclusion was used to handle the missing data for the covariates education level (*n* = 45), income (*n* = 34), gravidity (*n* = 104), physical activity use during pregnancy (*n* = 4), and STAI-state (*n* = 10).

In sensitivity analyses, additional adjustment for the day of recall, smoking during pregnancy, pre-pregnancy BMI, and omitting under-reporters was investigated. Statistical significance was considered at *p* < 0.05 and Stata version 14.2 (StataCorp LLC, College Station, TX, USA) was used for all of the analyses.

## 3. Results

### 3.1. Participants’ Characteristics

Good sleepers, as compared to poor sleepers, were sleeping longer and were more physically active, were less likely to be of Malay descent, and were less often anxious (Table 1). Good sleepers were not different to poor sleepers with regard to their age, total energy intake, education level, household income, gestational diabetes, parity, alcohol use during pregnancy, and pre-pregnancy BMI, physical activity level, and alcohol use. The mean (SD) HEI-SGP score for our study sample was 53.4 (13.8) out of the maximum of 100 points. 

### 3.2. Sleep Quality

Good sleepers, as compared to poor sleepers, reported better diet quality (54.6 vs. 52.0 points, *p* = 0.032) and greater adherence to the VFR pattern (0.03 vs. −0.15, *p* = 0.039; Table 2). These associations attenuated when additionally adjusting for anxiety. Adherence to the SFN pattern was significantly lower in the good sleepers as compared to the poor sleepers (−0.14 vs. 0.03, *p* = 0.024), which remained significant when including anxiety scores to the model (*p* = 0.018). No association was observed between sleep quality and the PCP pattern. 

Good sleepers consumed fewer discretionary calories, such as caloric beverages and local cakes as compared to poor sleepers (49.0 vs. 63.5 kcal, *p* = 0.073), but this association disappeared when additionally adjusting for anxiety scores. No association was observed between sleep quality and night-time fasting interval, the frequency of eating, or percentage calories consumed at night-time (Table 2).

### 3.3. Sleep Duration

Short sleep duration (<6 h) was not associated with diet quality, nor with any of the dietary patterns (Table 3). In addition, no associations were observed between sleep duration, and any of the eating behaviors.

### 3.4. Sensitivity Analyses

Additional adjustment for the day of recall, smoking during pregnancy, pre-pregnancy BMI, or omitting those that reported low-energy intakes (kcal < 500 [31]) did not alter our results (results not shown). Although significant interactions between sleep and depression and anxiety were observed, stratification was not possible due to a low number of participants with probable major depression (*n* = 45) or probable state anxiety (*n* = 103).

## 4. Discussion

Our results showed that good sleep quality was associated with better diet quality, as denoted by greater HEI-SGP scores and greater adherence to the VFR pattern, although these associations attenuated when taking anxiety scores into account. After additional adjustment for anxiety, good quality sleepers showed to be associated with lesser adherence to the SFN pattern in this cohort of pregnant women. No association was observed between sleep duration and dietary intake of dietary behaviors. 

Only a few other studies examined the relationship of sleep quality with diet. Similarly to our positive findings of sleep quality with diet quality and the VFR pattern, fewer insomnia symptoms have been reported with greater adherence to the Mediterranean diet [32]. The Mediterranean diet is characterized by high intakes of vegetables, legumes, fruits and cereals, fish, a moderate intake of alcohol, and low intakes of dairy, and saturated fatty acids, and is considered a healthy diet [33]. Moreover, a study investigating single food groups found that individuals with good sleep quality, defined as PSQI-Japan score ≤3, had significantly higher total vegetable and rice intakes in middle-aged Japanese females [34]. Another study in Japanese men and women further confirmed that a high rice intake was significantly associated with a 46% lower risk of poor sleep quality [35]. Suggested explanations were the high glycemic load of rice and the high content of melatonin of rice, which may favor good sleep [35]. In contrast to our findings, Chang et al. reported no associations between sleep quality using the PSQI and fat, and fruit and vegetable intakes among American overweight and obese pregnant women [18]. However, this study only examined women who were already overweight or obese and did not study overall diet or eating behaviors. Also, Cheng et al. [36] and Stern et al. [37] observed no associations between diet quality assessed by the ‘Alternate-HEI’ score and probable insomnia among American males or the women health initiative insomnia rating scale among postmenopausal women, respectively. 

We observed an inverse association between the SFN pattern and sleep quality. Previously, we showed that participants who adhered highly to the SFN pattern had higher intakes of energy, protein, and fat, and lower intakes of carbohydrates, and dietary fiber [24]; nutrients that have been suggested to be inversely associated with sleep [4,10]. Additionally, a study in Japanese participants found that the food group noodles was, independently from other carbohydrate sources as rice and bread, associated with poorer sleep quality [35].

While differences in diet quality scores between good and poor sleepers may appear moderate, there is evidence to demonstrate that the associations between diet quality and all-cause and cause-specific mortality are dose-response relationships [38], suggesting that any improvement in diet quality will result in some health improvements. Furthermore, even moderate changes in diet quality have been shown to decrease the risk of death meaningfully [39]. This evidence from other diet quality indices suggests that our rather small mean differences in diet between good/poor sleepers may be of clinical relevance.

We showed no association between sleep duration and dietary intake, which corroborates with two studies among non-pregnant women and men, [13,40], but not with other studies [8,9,36,37,41]. This discrepancy might be explained by the differences in methodology of the various studies, for example in definitions of short sleep duration (varying between <5 and <8 h), of diet quality indices (e.g., Alternate-HEI, HEI, Diet Quality Index for Adolescents with Meal index), or a different set of confounders included in the statistical models. Another explanation might be the difference in the cause of sleep problems in our population when compared to non-pregnant study samples [17,42].

Evidence for the relationship between sleep and eating behaviors is very limited, although it is important as it can provide important context for future interventions. We observed no association between sleep quality or sleep duration with any of the dietary behaviors. In contrast, Kant et al. found that calories consumed from all snacks decreased with increasing sleep duration in an adult American population sample [12], however, sleep quality was not studied. Similar to our findings, they did not find an association between the number of eating episodes and sleep duration. More studies are needed to further elucidate the relationship between sleep and eating behaviors, also looking into sleep quality.

Mental well-being is linked to poor sleep and has been associated with dietary intake [43], and might thus modify or confound the relation between sleep and diet. For this reason, we excluded the women who had probable major depression (EPDS scores ≥ 15; *n* = 45) and we additionally adjusted for STAI-state scores in model 2. Hence, our results showed that even for women without major mental health issues, there is a relation between sleep quality and dietary intake. 

Both sleep and diet are paramount for overall health and mental well-being [4,24], and this might be even more important in pregnant women. Sleep and diet during pregnancy can affect gestational weight gain, risk of delivery complications, and has an impact on the offspring’s health in later life [44,45,46]. On top of this, it is well-known that sleep quality and sleep duration are often compromised in pregnant women and this deteriorates over the course of pregnancy [15,16]. It is therefore crucial to make pregnant women aware of these risks and to improve their sleep practices. Health practitioners who are involved in antenatal care should be alert for sleep problems during pregnancy and educate women on healthy sleep hygiene practices and possible behavioral changes or therapies [47]. 

The strengths of this study are the inclusion of a relatively large number of apparently healthy pregnant women, and the use of dietary patterns that enabled the examination of synergistic effects of nutrients. Furthermore, we assessed sleep quality that includes both quantitative aspects of sleep, such as sleep duration and qualitative subjective aspects of sleep, whereas most studies only investigate sleep duration. The PSQI questionnaire, moreover, enabled us to differentiate between good and poor sleepers. 

However, we also need to acknowledge the limitations of our study. Sleep and dietary intake were assessed at a similar point in time, through which no conclusions can be drawn on the causality of the association. Trials have shown associations between sleep and diet in both directions (sleep affecting dietary intake and vice versa) [1,10], which suggest a bi-directional relationship. Secondly, sleep was assessed by self-report on a subjective scale, which may induce misreporting and thereby attenuation of our results. Objective measurements, such as polysomnography or multiple actigraphs, could have strengthened our associations. Nevertheless, the PSQI was previously validated against objective sleep measures [27,28]. Furthermore, we excluded quite a large number of participants who did not complete the sleep questionnaire (*n* = 451) that could have led to selection bias. In general, the participants who completed the sleep questionnaire had higher household incomes, education levels, were more often employed, married, and adopted healthier lifestyles as compared to those who had incomplete sleep questionnaire (results not shown). Thirdly, dietary intake was assessed with only one 24-h recall and is thus not representing usual intake. Two or more 24-h recalls are recommended to capture some of the variability in dietary intake depending on the dietary habits of the population under study [48]. Though, we think that our 24-h recall adequately represented the typical diet of our participants, as showed by the moderate correlations between the emerged dietary patterns from 24-h recall and those based on three-day food diaries that were administered in a subsample of the GUSTO cohort (*n* = 255; Pearson’s correlation r_VFR_ = 0.48, r_SFN_ = 0.52). 

## 5. Conclusions

We are the first to show that good sleep quality was accompanied by a significantly lesser adherence to the seafood-noodle pattern during pregnancy and borderline significantly better diet quality and greater adherence to the vegetable-fruit-rice pattern. No associations were observed for sleep duration with diet or eating behaviors. While we cannot conclude the direction of our associations, our findings suggest the value of increasing awareness of the importance of good sleep quality and the promotion of healthy sleep practices in future mothers. Better sleep quality may not only improve dietary and subsequent health improvements for maternal and offspring health outcomes [24,44], but better sleep quality itself has also been positively associated with less health issues [45,46]. Future studies should investigate the causality of the relationship between dietary patterns and sleep quality in pregnant women. Moreover, future study should also investigate which aspects of poor sleep, besides sleep duration, are important in relation to diet and eating behaviors to further unravel this relationship. 

## Figures and Tables

**Table 1:** Participant characteristics among poor versus good sleepers *.

	Poor Sleep Quality (PSQI > 5)	Good Sleep Quality (PSQI ≤ 5)	*p* Value
N	271	226	
Age (y)	30.8 (4.5)	30.7 (5.0)	0.942
Pre-pregnancy BMI (kg/m^2^)	22.8 (4.2)	22.5 (4.5)	0.463
Sleep duration (hours)	6.6 (1.4)	8.0 (1.2)	<0.001
Energy intake (kcal)	1902 (551)	1875 (573)	0.596
Education			0.130
Primary/secondary	70, 25.8%	48, 21.2%	
Postsecondary	37, 13.7%	22, 9.7%	
University	164, 60.5%	156, 69.0%	
Household income			0.171
S$0–1999	37, 13.7%	21, 9.3%	
S$2000–5999	79, 29.2%	59, 26.1%	
≥S$6000	155, 57.2%	146, 64.6%	
Ethnicity			0.018
Chinese	127, 46.9%	137, 60.6%	
Malay	87, 32.1%	52, 23.0%	
Indian	56, 20.7%	37, 16.4%	
Pre-pregnancy physical active	171, 63.6%	146, 64.6%	0.811
Physical active during pregnancy	61, 22.5%	74, 32.7%	0.011
pre-pregnancy alcohol use	168, 62.0%	140, 62.0%	0.992
Alcohol use during pregnancy	3, 1.1%	5, 2.2%	0.332
First child	117, 43.2%	106, 46.9%	0.405
Gestational Diabetes	55, 21.8%	36, 16.9%	0.182
Probable anxiety	76, 28.0%	27, 12.0%	<0.001

* Mean (SD) or *n*, %; PSQI: Pittsburgh sleep questionnaire, BMI: Body mass index.

**Table 2:** Adjusted means and 95% CI of dietary patterns and eating behaviors by categories of sleep quality among 497 pregnant women of the Growing Up in Singapore Towards Healthy Outcomes (GUSTO) cohort.

	Model 1					Model 2				
	Poor Sleep Quality (*n* = 271)		Good Sleep Quality (*n* = 226)		*p* for Difference	Poor Sleep Quality (*n* = 271)		Good Sleep Quality (*n* = 226)		*p* for Difference
	Mean	95% CI	Mean	95% CI		Mean	95% CI	Mean	95% CI	
Diet quality (HEI-SGP scores)	52.0	50.4, 53.6	54.6	52.9, 56.3	0.032	52.4	50.8, 54.0	54.5	52.7, 56.2	0.096
VFR	−0.15	−0.26, −0.04	0.03	−0.09, 0.15	0.039	−0.13	−0.24, −0.02	0.02	−0.10, 0.14	0.087
SFN	0.03	−0.07, 0.13	−0.14	−0.25, −0.03	0.024	0.03	−0.07, 0.13	−0.15	−0.26, −0.04	0.018
PCP	0.03	−0.09, 0.14	0.00	−0.13, 0.12	0.749	0.04	−0.08, 0.16	−0.01	−0.14, 0.12	0.621
**Eating behaviors**										
Night-time fasting interval (h)	9.83	9.64, 10.00	9.86	9.66, 10.06	0.819	8.80	9.62, 9.99	9.84	9.63, 10.05	0.800
Frequency of consumption occasions	4.12	3.99, 4.25	4.26	4.11, 4.40	0.169	4.14	4.01, 428	4.27	4.12, 4.42	0.228
Discretionary calories (kcal) ^†^	382.6	346.0, 419.2	330.5	290.3, 370.6	0.063	375.9	3387, 413.0	338.6	297.7, 379.4	0.198
Night-time eating (TE%)	33.9	31.8, 36.1	32.7	30.3, 35.0	0.476	33.7	31.5, 35.8	33.1	30.7, 35.5	0.732

HEI-SGP: Healthy eating index for pregnant women in Singapore, VFR: vegetable, fruit and white rice pattern, SFN: seafood, fish and noodle pattern, PCP: pasta, cheese and processed meat pattern, TE%: total energy percent; Model 1: Adjusted for alcohol during pregnancy, physical activity during pregnancy, household income, education level, ethnicity, total energy intake, age, gravidity; Model 2: Adjusted for model 1 and additionally for anxiety scores; ^†^ Energy from discretionary foods included caloric beverages (excluding plain water, diet sodas, and unsweetened coffee, tea and cow’s milk), local cakes, desserts and snacks.

**Table 3:** Adjusted means and 95% CI of dietary patterns and eating behaviors by categories of sleep duration among 497 pregnant women of the GUSTO cohort.

	Model 1					Model 2				
	Sleep Duration ≤ 6 h (*n* = 134)		Sleep Duration > 6 h (*n* = 363)		*p*	Sleep Duration ≤ 6 h (*n* = 134)		Sleep Duration > 6 h (*n* = 363)		*p*
	Mean	95% CI	Mean	95% CI		Mean	95% CI	Mean	95% CI	
Diet quality (HEI-SGP scores)	52.3	50.1, 54.6	53.5	52.1, 54.8	0.405	53.0	50.8, 55.3	53.5	52.1, 54.9	0.723
VFR	−0.15	−0.30, 0.01	−0.04	−0.13, 0.06	0.245	−0.13	−0.28, 0.03	−0.04	−0.13, 0.06	0.353
SFN	0.01	−0.13, 0.15	−0.07	−0.15,0.02	0.378	0.00	−0.15, 0.14	−0.07	−0.15, 0.02	0.449
PCP	−0.02	−0.18, 0.14	0.03	−0.07, 0.13	0.627	−0.01	−0.18, 0.15	0.03	−0.07, 0.13	0.659
**Eating behaviors**										
Night-time fasting interval (h)	9.75	9.49, 10.01	9.87	9.72, 10.03	0.418	9.7	9.5, 10.0	9.9	9.7, 10.0	0.365
Frequency of consumption occasions	4.11	3.92, 4.30	4.21	4.10, 4.32	0.368	4.15	3.96, 4.33	4.22	4.11, 4.33	0.514
discretionary calories (kcal) ^†^	361.6	309.1, 414.1	357.9	326.3, 389.6	0.908	358.4	306.1, 410.7	359.1	327.6, 390.6	0.982
Night-time eating (TE%)	33.2	30.2, 36.3	33.4	31.6, 35.3	0.920	33.1	30.1, 36.2	33.5	31.6, 35.3	0.854

HEI-SGP: healthy eating index for pregnant women in Singapore, VFR: vegetable, fruit and white rice pattern, SFN: seafood, fish and noodle pattern, PCP: pasta, cheese and processed meat pattern, TE%: total energy percent; Model 1: Adjusted for alcohol during pregnancy, physical activity during pregnancy, household income, education level, ethnicity, total energy intake, age, and gravidity; Model 2: Adjusted for model 1 and additionally for anxiety scores; ^†^ Energy from discretionary foods included caloric beverages (excluding plain water, diet soda, and unsweetened coffee, tea and cow’s milk), local cakes, desserts and snacks.

## Supplementary Materials

The following are available online at http://www.mdpi.com/1660-4601/14/11/1409/s1, Table S1: Factor loading matrix for the 3 major dietary patterns identified by using 24-h recalls in the complete GUSTO cohort (*n* = 1170) and subsample with complete data on sleep quality (*n* = 497).
