# Supplementary material for: Sleep and Dietary Patterns in Pregnancy: Findings from the GUSTO Cohort
Source: Int J Environ Res Public Health. 2017 Nov 17;14(11):1409. doi: 10.3390/ijerph14111409 (PMC5708048; doi:10.3390/ijerph14111409)
Supplement: Supplementary file 1 [file ijerph-14-01409-s001.pdf]

Table S1. Factor loading matrix for the 3 major dietary patterns identified by using 24-h recalls in the complete GUSTO cohort (n=1170) and subsample with complete data on sleep quality (n=497)

Food or Food groups	Vegetable, Fruit and white Rice diet (VFR)		Seafood and Noodle diet (SfN)		Pasta, Cheese and Processed meat (PCP)	
	Complete sample	Subsample	Complete sample	subsample	Complete sample	subsample
Cruciferous, leafy-green and dark-yellow vegetables	0.53	0.39	-	-	-	-
Other vegetables[2]	0.44	0.32	-	-	-	-
Fried potatoes	-0.42	-0.33	-	-	-	-
Fruits	0.39	-	-	-	-	-
Hamburger	-0.34	-0.30	-	-	-	-
White rice	0.34	0.26	-0.28	-0.24	-	-
Carbonated drinks	-0.32	-0.26	-	-	-	-
Flavoured rice	-0.30	-0.24	-	-	-	-
Whole-grain bread	0.29	0.28	-	-	-	-
Sweetened drinks[3]	-0.26	-0.26	-	-	-	-
Red meat and poultry(Deep fried/in curry)	-0.25	-0.25	-	-	-	-
Non-fried fish	0.22	-	-	-	-	-
Milk-based drinks	-0.21	-	-	-	-	-
Nuts and seeds	0.21	-	-	-	-	-
Soup	-	-	0.52	0.54	-	-
Ethnic bread[4]	0.21	0.23	-0.43	-0.28	-	-
Fish and seafood products	-	-	0.43	0.36	-	-
Noodles (in soup)	-	-	0.41	0.45	-	-
Legumes and pulses	0.21	0.21	-0.41	-0.22	-	-
Flavoured noodles	-	-	0.39	-	-	-
Low fat red meat	-	-	0.35	0.32	-	-
Seafood	-	-	0.29	-	-	-
Curry based gravies	-	-	-0.27	-	-	-
Soya sauce based gravies	-	-	0.26	-	-	-
Other grains[5]	-	-	-0.23	-	-	-
Eggs	-	-	0.21	-	-	-
Local savoury snacks	-	-	0.21	-	-	-
Pasta	-	-	-	-	0.81	1.00
Tomato based gravies	-	-	-	-	0.69	0.56
Cream based gravies	-	-	-	-	0.46	0.32
Cheese	-	-	-	-	0.45	-
Processed meats	-	-	-	-	0.27	-
Correlation between complete and subsample	0.95		0.94		0.32	

[1] values∣< 0.20∣ were not listed in the table for simplicity; [2] Vegetables other than cruciferous, leafy-green and dark-yellow vegetables; [3] Non-carbonated, cordial and fruit drinks; [4] Chinese steamed bun, tortilla, idli, puri , thosai, chapati, naan; [5] Uppuma, millet, corn, glutinous rice.
